# The Superior Cerebellar Artery: Variability and Clinical Significance

**DOI:** 10.3390/biomedicines11072009

**Published:** 2023-07-17

**Authors:** Mikołaj Malicki, Bartosz M. Szmyd, Ernest J. Bobeff, Filip F. Karuga, Michał M. Piotrowski, Dawid Kościołek, Sora Wanibuchi, Maciej Radek, Dariusz J. Jaskólski

**Affiliations:** 1 Department of Neurosurgery, Spine and Peripheral Nerves Surgery, Medical University of Lodz, Zeromskiego St. 113, 90-549 Lodz, Poland; mikolaj.malicki@stud.umed.lodz.pl (M.M.); maciej.radek@umed.lodz.pl (M.R.); 2 Department of Neurosurgery and Neuro-Oncology, Medical University of Lodz, Barlicki University Hospital, Kopcinskiego St. 22, 90-153 Lodz, Poland; bartoszmyd@gmail.com (B.M.S.); michal.piotrowski@umed.lodz.pl (M.M.P.); dariusz.jaskolski@umed.lodz.pl (D.J.J.); 3 Department of Pediatrics, Oncology and Hematology, Medical University of Lodz, Sporna St. 36/50, 91-738 Lodz, Poland; 4 Department of Sleep Medicine and Metabolic Disorders, Medical University of Lodz, Mazowieka St. 6/8, 92-251 Lodz, Poland; filip.karuga@umed.lodz.pl; 5 Central Teaching Hospital, Medical University of Lodz, Pomorska St. 251, 92-208 Lodz, Poland; dawid.kosciolek@stud.umed.lodz.pl; 6 The Faculty of Medicine, Aichi Medical University, Nagakute 480-1195, Japan; wanibuchi.sora@gmail.com

**Keywords:** superior cerebellar artery, anatomical variants, neurovascular compression syndrome, trigeminal neuralgia, hemifacial spasm, oculomotor compression syndrome

## Abstract

The superior cerebellar artery (SCA) arises from the distal part of the basilar artery and passes by the oculomotor, trochlear, and trigeminal nerves. SCA is known to play a crucial role in the development of trigeminal neuralgia. However, due to its anatomical variability, it may also trigger other neurovascular compression (NVC), including hemifacial spasm, oculomotor nerve palsy, and ocular neuromyotonia. Additionally, it may be associated with ischemic syndromes and aneurysm development, highlighting its clinical significance. The most common anatomical variations of the SCA include duplication, a single vessel origin from the posterior cerebral artery (PCA), and a common trunk with PCA. Rarely observed variants include bifurcation and origin from the internal carotid artery. Certain anatomical variants such as early bifurcation and caudal course of duplicated SCA trunk may increase the risk of NVC. In this narrative review, we aimed to examine the impact of the anatomical variations of SCA on the NVCs based on papers published in Pubmed, Scopus, and Web of Science databases with a snowballing approach. Our review emphasizes the importance of a thorough understanding of the anatomical variability of SCA to optimize the management of patients with NVCs associated with this artery.

## 1. Introduction

### 1.1. Background on the Superior Cerebellar Artery

**Figure 1:**
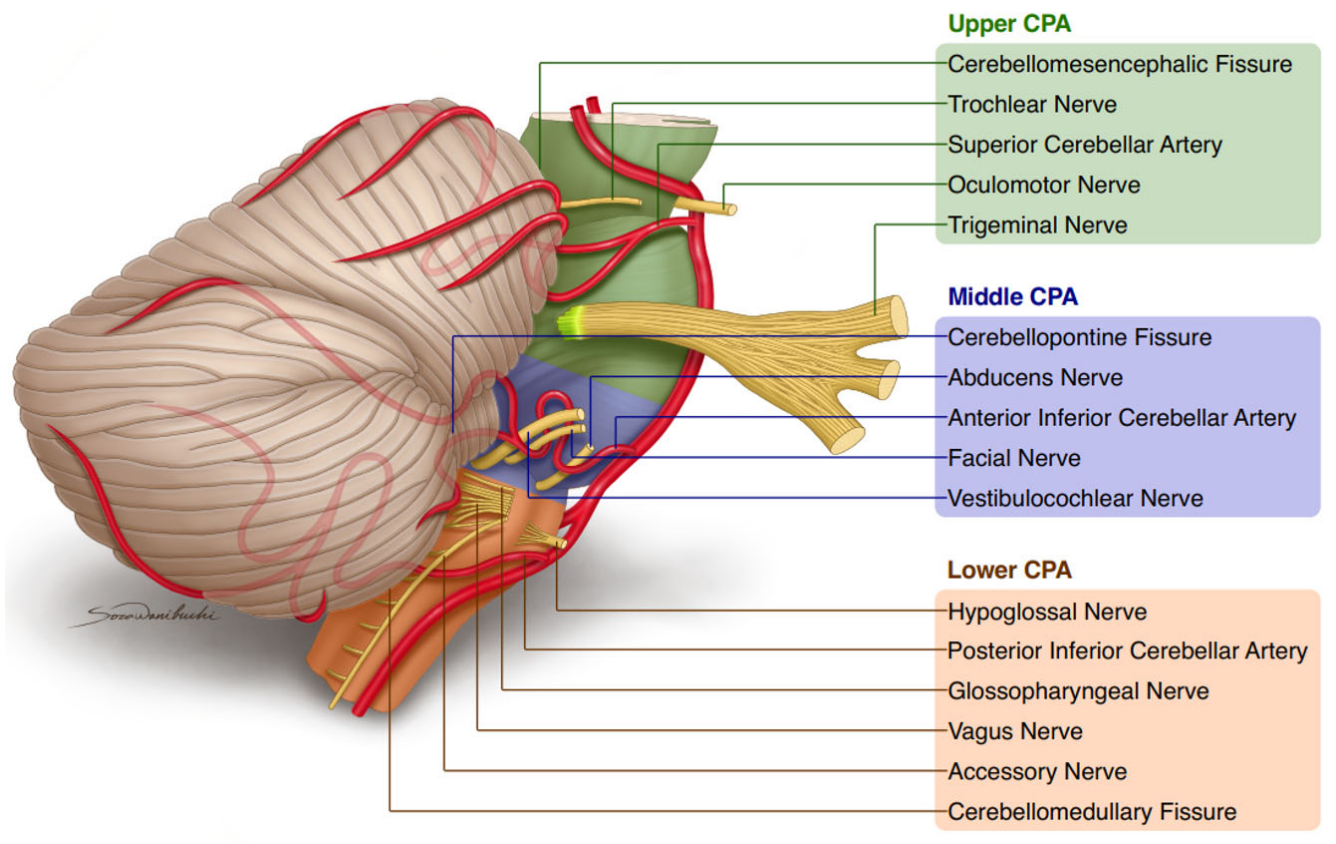
Summary of basic knowledge about superior cerebellar artery. Legend: CPA—cerebellopontine angle.

The superior cerebellar artery (SCA) is a branch of the distal part of the basilar artery (BA), passing in a posterolateral direction. During this course, the following segments are distinguished: anterior pontomesencephalic, lateral pontomesencephalic, cerebellomesencephalic, and cortical (see Figure 1). SCA provides blood supply for the pons, cerebral peduncle, the upper part of the cerebellum, and cerebellar peduncles [1,2].

### 1.2. Neurovascular Compression Syndromes

Neurovascular compression (NVC) syndromes are a group of disorders with a complex etiology. Numerous mechanisms have been proposed in their pathophysiology, but none have provided a sufficient explanation. The most likely chain of events involves the mechanical compression on the cranial nerve root entry/exit zone (REZ) by a pulsating vessel, leading to demyelination, disturbances in ion channels, and hyperexcitability of the nucleus [3,4,5,6,7,8,9]. Nevertheless, the mere presence of direct contact between these structures is insufficient for the diagnosis of NVC. The abnormal course of a vessel may also play a role in the development of these syndromes [10].

### 1.3. Importance of Understanding Anatomical Variations of the SCA

The cerebellopontine angle (CPA) can be divided into three parts: upper, middle, and lower (Figure 1) [11,12,13]. The upper CPA contains SCA and the cranial nerves: oculomotor, trochlear, and trigeminal. The middle CPA contains the anterior inferior cerebellar artery (AICA) and the cranial nerves: abducens, facial, and vestibulocochlear [14]. The lower CPA contains the posterior inferior cerebellar artery (PICA) and the cranial nerves: glossopharyngeal, vagus, accessory, and hypoglossal.

SCA, located in the upper CPA, is associated with trigeminal neuralgia (TN) and oculomotor compression syndromes such as oculomotor nerve palsy (ONP) and ocular neuromyotonia (NMT) [15,16,17,18,19]. However, it has also been described in conflict with the REZ of facial nerve leading to hemifacial spasm (HFS) [20]. These diverse clinical manifestations of NVC syndromes triggered by SCA highlight the importance of its anatomical variability [21].

SCA may be also associated with strokes and aneurysms [22,23]. SCA strokes are more frequently observed among patients with hypertension, cardiopathies, arrhythmias, hyperlipidemia, and diabetes, and those who smoke or use oral contraceptives [24]. Typical symptoms include nausea, vomiting, dizziness, tinnitus, headache, ataxia, and dysarthria [25]. SCA aneurysms account for less than 2% of intracranial aneurysms [26]. In a series of 36 SCA aneurysms treated by coiling, 65% presented with subarachnoid hemorrhage and 14% of patients had multiple intracranial aneurysms [26]. Interestingly, there were no episodes of rebleeding during the median clinical follow-up of 44.5 months (range 4–103 months, 118 patient-years) [26]. The only two cases of SCA aneurysm treated at our departments of neurosurgery in the last 10 years are provided in the Supplementary Material.

## 2. Anatomy of the Superior Cerebellar Artery

SCA typically originates as a single vessel from the distal part of BA [27,28,29]. After ~18.5 mm from the origin, near its maximal caudal descent, SCA bifurcates into two major trunks of similar diamaters: rostral and caudal [30]. The rostral trunk terminates by giving hemispheric, vermian, and perforating arteries. The caudal trunk provides blood supply through hemispheric, marginal, and perforating arteries.

Four segments can be distinguished on the SCA course: S1: anterior pontomesenphalic, S2: lateral pontomesenphalic, S2: cerebellomesoencephalic, and S4: cortical ones [31]. The anterior pontomesenphalic is the first SCA part passing inferior to the oculomotor nerve [28]. The lateral pontomesenphalic segment begins at the anterolateral margin of the brainstem and frequently dips caudally onto the lateral side of the upper pons [32]. Its caudal loop projects forward and may reach the oculomotor nerve at its REZ [30]. The trochlear nerve is frequently observed in the midportion of this segment. This segment passes into the cerebellomesencephalic segment at the anterior margin of the cerebellomesencephalic fissure [30]. In this segment, SCA branches enter the shallowest part of the fissure located posterosuperior to the REZ of the trigeminal nerve and course medial to the tentorial edge with its branches intertwined with the trochlear nerve [32]. The last, cortical segment, comprises branches located distally to the cerebellomesencephalic groove, which supply the cortex of the vermis and cerebellar hemisphere [30]. SCA provides blood supply for the pons, cerebral peduncle, the upper part of the cerebellum, and cerebellar peduncles [1,2,28].

## 3. Neurovascular Compression Syndromes Related to the Superior Cerebellar Artery

### 3.1. Trigeminal Neuralgia

TN patients may experience even 10–50 pain attacks per day [33,34,35]. A single attack may be described as electric-like, sharp, severe, and superficial pain. It can be accompanied by unintentional spasms of the facial muscles [36]. Therefore, TN significantly impacts a patient’s quality of life and may lead to psychological distress, anxiety, depression, and even suicide attempts [37,38,39,40]. Its prevalence is difficult to estimate and ranges from 0.03% to 0.3% [41,42,43,44]. It is higher in women over 40 years of age [44].

NVC represents the dominant cause of TN [45]. In the 1930s, Walter Dandy noted that the sensory roots of the trigeminal nerve are usually touched and/or modeled by an artery [46]. Several vessels may be responsible for NVC leading to TN (Table 1), primarily SCA (up to 90%) and anterior inferior cerebellar artery (AICA, 7–23.6%). The influence of their anatomical variants on the risk, prevalence, and severity of NVC is poorly understood. It was reported that tortuous and elongated SCA can increase the risk of TN [47]. In one MRI-based study duplicated SCAs were involved in TN [48]. The authors suggested that if one of the trunks takes a caudal course, duplication or early bifurcation of SCA may increase the risk of TN [48]. Rarely, TN is due to NVC with other vessels (including veins, e.g., superior petrosal venous complex [49] and arteries transfixing the sensory root of trigeminal nerve [50]) and/or arteriovenous malformation (AVM) arising from a dilated SCA, AICA, or posterior meningeal artery with venous drainage to the basal vein of Rosenthal, then posing a diagnostic challenge [51,52,53]. In these cases, SCA can act either as a compressing vessel or as a vessel supplying the AVM associated with TN [54,55,56].

Differential diagnosis of TN should encompass the following conditions: trauma [36], viral infections (e.g., SARS-CoV-2, Herpes Zoster) [57,58], multiple sclerosis [59], and intracranial malignancy [60]. In such cases, facial pain may be accompanied by other neurological symptoms as an effect of underlying pathology [61].

### 3.2. Oculomotor Compression Syndromes

The oculomotor nerve originates from the midbrain and passes laterally between the posterior cerebral artery (PCA) and SCA [62]. Both arteries provide their blood supply in the cisternal (initial) parts [63]. In further subchapters, we presented available information about NVCs among patients with oculomotor nerve palsy and ocular neuromyotonia. As only single cases of successful MVD in these patients were reported up to date, it is questionable if these entities are related to NVC.

#### 3.2.1. Oculomotor Nerve Palsy

ONP may be due to NVC of the oculomotor nerve by SCA, mainly characterized by diplopia and ipsilateral ptosis [15,16,17,64,65]. Differential, and more common, diagnoses encompass aneurysms (especially those located in the BA tip, BA-SCA junction, posterior communicating artery, and cavernous segment of internal carotid artery), diabetes mellitus, trauma, brain tumors, infarction, and central nervous system infections [17,27]. A case of NVC of the oculomotor nerve between the atherosclerotic PCA and SCA was described [16]. The resolution of symptoms after microvascular decompression (MVD) indicates that in this case, NVC has triggered ONP. It is worth noting that in this patient, the MRI scan did not show evident NVC, and the diagnosis was confirmed intraoperatively [16]. One study described the NVC of the superomedial portion of the oculomotor nerve between the duplicated SCA and PICA. It was revealed on MRI and manifested by isolated pupillary dilation, but no information on treatment (including MVD) was provided [66]. Similarly, transient ONP due to NVC with flattening and elevation of the oculomotor nerve over a duplicated SCA and no other pathologies on imaging was described [15]. In this case, there was no information about treatment (including MVD).

**Table 1:** Superior cerebellar artery as key vessel leading to NVC-related trigeminal neuralgia. Studies are arranged chronologically.

Anatomical Structure	Zorman & Wilson 1984 N = 118 [67]	Sindou et al., 1994 N = 322 [68]	Barker et al., 1996 N = 1204 [69]	Sindou et al., 2002 N = 579 [70]	Li et al., 2004 N = 62 [71]	Sekula et al., 2009 N = 14 [72]	Lorenzoni et al., 2012 N = 100 [4]
SCA	83.3%	90%	75%	88%	58%	57%	71%
AICA	15.5%	23.6%	10%	25.1%	38.7%	7%	11%
Others	PICA (1.1%)	BA (3.2%)	Small arteries (14%) Others **	BA (3.5%)	PICA (12.9%)	Small arteries (14%)	BA (2%) VA (2%)

Legend: AICA—anterior inferior cerebellar artery, BA—basilar artery, PICA—posterior inferior cerebellar artery, SCA—superior cerebellar artery, Small arteries—small diameter, unnamed arteries. **—VA (2%), PICA (1%), BA (1%), Labyrinthine artery (<1%).

#### 3.2.2. Ocular Neuromyotonia

Ocular neuromyotonia (NMT) is another disorder potentially associated with NVC of the cisternal portion of SCA or less frequently PCA on the oculomotor nerve [18,19]. It is characterized by recurrent brief episodes of diplopia due to tonic extraocular muscle contraction and chronically damaged oculomotor nerve [73]. In such cases, typical MRI findings and symptom relief after MVD supports the diagnosis of NVC [18]. Based on MRI, the ocular NMT was also described as a result of NVC by elongated SCA [19].

Most of the literature base the diagnosis of NVC solely on radiological examinations, which carries the risk that the observed NVCs were nothing but incidental findings, not the proper cause of the symptoms [19,48,74]. The NVC hypothesis can be confirmed unequivocally only by the resolution of symptoms after MVD [18,19].

### 3.3. Hemifacial Spasm

HFS results from NVC of the facial nerve’s REZ and presents with intermittent twitching of muscles starting from the orbicularis oculi muscle [20,75]. Involuntary unilateral clonic and tonic movements of facial expression muscles may also occur [76,77]. Prevalence is approximately 10 per 100,000 [78,79]. The REZ of the facial nerve could be compressed by AICA, posterior inferior cerebellar artery (PICA), SCA, and vertebral artery (VA) [20,80,81,82]. Particularly, lateral deviation of VA increased the risk of HFS [20,80,81]. The exact role of SCA anatomical variants in HFS is not well established. Differential diagnosis should encompass: infections (e.g., otitis media, tubercular meningitis, COVID-19) [83,84], cerebellopontine angle tumors, brainstem damage, etc. [49,50,51].

## 4. Anatomical Variations of the Superior Cerebellar Artery

We reviewed studies that used various methods for the detection of the SCA anatomical variants (Table 2): anatomical dissection of cadaveric brain specimens [1,85,86,87,88,89,90], computed tomography angiography [91,92], and MRI angiography [48].

**Figure 2:**
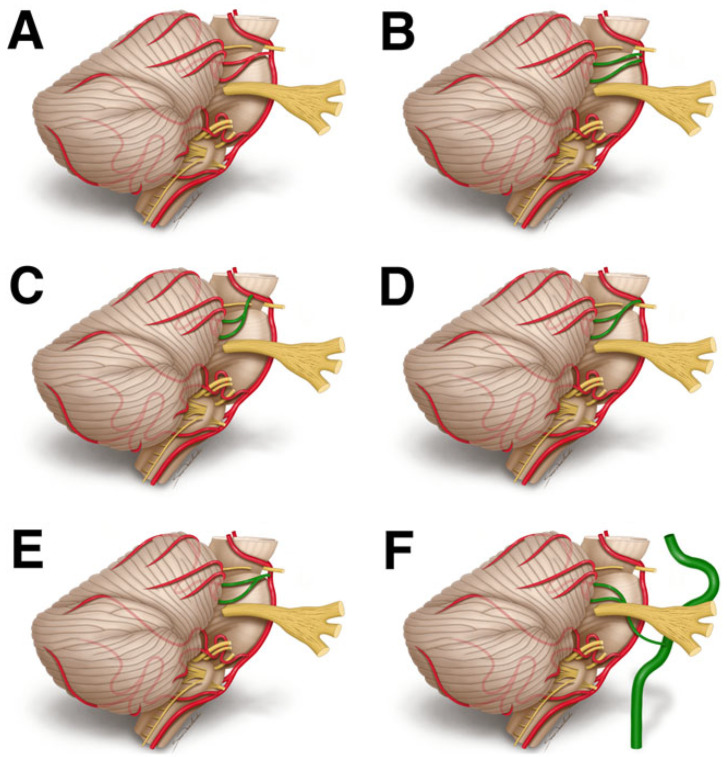
The possible SCA course: (**A**) typical course, (**B**) SCA duplication, (**C**) single vessel origin from the posterior cerebral artery, (**D**) common trunk with the posterior cerebral artery, (**E**) bifurcation, (**F**) origin from the internal carotid artery. Note, that (**D**) is a type of (**C**), in which the P1 gives off the SCA and eventually narrows (we decided to distinguish this type as it was observed in previous papers).

The reported variants included: (1) SCA duplication [1,48,85,86,88,89,90,91,92], (2) SCA originating as a single vessel from PCA [1,88,89,90,91,92], and (3) SCA creating a common trunk with PCA [1,48,87,89,90,91,92], (4) early SCA bifurcation [48,91], and (5) SCA originating from the internal carotid artery (ICA, Figure 2) [48].

Duplication: Duplication frequency varies from ~3% to ~30% [86,92]. The frequency of the left and right duplication is similar [48,86,92]. The bilateral variant was observed in 0.9–5% [1,86]. There was a single case of an 84-year-old woman with SCA duplication potentially associated with cerebellar infarction [23]. SCA triplication is sevenfold less frequent [90].

Other origination sites: SCA usually originates from the distal part of BA, however, it may also originate from the PCA or as a common trunk with PCA from BA. The first variant is observed in 1.88–15%, with bilateral occurrence in 1.2–5% of cases [1,90]. The second one was described in 1–5.93%, with bilateral occurrence in 0.7–1% of cases [87,92].

Early bifurcation: SCA commonly bifurcates into two major trunks, namely rostral and caudal, at a distance of 0.6–34.0 mm from its origin. When the bifurcation occurs at the proximal anterior pontomesencephalic segment, it is referred to as early bifurcation [48]. This variant has been reported in 3–9.4%, with bilateral occurrence in up to 0.9% of cases [48,91].

**Table 2:** Prevalence of SCA anatomical variations. Studies are arranged chronologically.

Type of the Variation	Percentage Calculated Based on the Total Number of Patients							Percentage Calculated Based on the Total Number of SCAs		
	Blackburn (1907) N = 220 [85]	Stopford (1916) N = 150 [86]	Caruso et al., (1991) N = 100 [87]	Uchino et al., (2003) N = 136 [48]	Pai et al., (2007) N = 25 [88]	Garcia-Gonzalez et al., (2012) N = 20 [1]	Pekcevik et al., (2013) N = 341 [91]	Krzyżewski et al., (2014) * N = 200 [92]	Ogeng’o et al., (2015) * N = 173 [89]	Kalaiyarasi and Chitra (2018) * N = 80 [90]
Typical variant (SCA bilaterally originating as a single vessel from the BA)	N.D.	N.D.	N.D.	82%	80%	35%	70.7%	89.43%	72.1%	75.6%
SCA originating from the PCA as a single vessel	N.D.	N.D.	N.D.	N.D.	4%	15%	4.7%	4.64%	2%	1.88%
SCA originating as a common trunk with PCA **	N.D.	N.D.	1%	4.4%	N.D.	40%	5%	5.93%	2.5%	3.13%
SCA originating from the ICA	N.D.	N.D.	N.D.	0.7%	N.D.	N.D.	N.D.	N.D.	N.D.	N.D.
Duplication of SCA originated from BA	3.61%	31%	N.D.	9.6%	16%	15%	20.5%	3.09%	21.3%	17.5%
SCA duplicated and originating from the PCA	N.D.	N.D.	N.D.	N.D.	N.D.	N.D.	0.3%	N.D.	2%	N.D.
Early bifurcation of the SCA	N.D.	N.D.	N.D.	3%	N.D.	N.D.	9.4%	N.D.	N.D.	N.D.
Absence of the SCA	N.D.	0.67%	N.D.	N.D.	N.D.	N.D.	N.D.	4%	N.D.	N.D.
Simultaneous duplication and common trunk of SCAs	N.D.	N.D.	N.D.	N.D.	N.D.	N.D.	1.8%	N.D.	N.D.	N.D.

Legend: ICA—internal carotid artery, N.D.—no data, PCA—posterior cerebral artery, SCA—superior cerebellar artery; *—these data are calculated regarding the total number of SCAs (not patients), **—a common trunk with *PCA* is an example of SCA originating from the PCA as a single vessel in which the P1 gives off the SCA and eventually narrows (we decided to distinguish this type as it was observed in previous papers).

Origin from the internal carotid artery is observed in less than 1% of cases, in which persistent trigeminal artery (PTA) supplies SCA [48]. This variant is known as Saltzman type II PTA. In such cases, the anterior pontomesencephalic segment is absent owing to the failure of fusion in the early embryonic stage [48].

## 5. The Approach of Better Visualization of Anatomical Variations in Diagnosis and Surgical Treatment

Proper patients qualification for treatment remains one of the most important steps of the diagnostic and therapeutic approach. The qualification for different invasive procedures (among others: peripheral trigeminal nerve branch procedures, percutaneous trigeminal rhizotomy, MVD, and stereoradiosurgery with gamma knife) for TN remains a subject of separate and still pending discussion and may be affected by the personal preferences of both patients and neurosurgeons. One of the leading indications for MVD in TN is the inability to achieve adequate medical control of TN trigeminal with ≥5 years of anticipated survival, without significant medical or surgical risk factors [93]. Furthermore, the knowledge of possible anatomical variants is crucial for achieving good surgical outcomes in SCA-related NVC syndromes, as confirmed by the relief of symptoms following MVD.

Neuroimaging enables the proper differential diagnosis in NVC patients. It allows us to rule out the entities such as brain tumors, aneurysms, arteriovenous malformations, and others. The success rate of MVD strongly depends on careful visualization of the nerve in its REZ during the surgery, not on radiological examination. Many NVCs, that can be observed intraoperatively are not seen in neuroimaging. On the other hand, visualization of contact between the nerve and vessel in the radiological examination without clinical symptoms is not sufficient for the diagnosis of NVCs. Therefore, the following subchapters discuss many issues of fairly limited direct clinical importance.

### 5.1. Magnetic Resonance Imaging

Meaney et al. used magnetic resonance angiography for NVC visualization in 1995 [94]. Several years later Naraghi et al. used the heavily T2-weighted sequences by constructive interference in steady-state [95]. Contemporary, the most applicable in the context of NVC detection are the combination of high-resolution 3D T2-weighted imaging with 3D time-of-flight angiography and 3D T1-weighted gadolinium-enhanced sequences [96].

As important as selecting the appropriate MR sequence is correct interpreting the imaging examination since, as has already been mentioned, mere visualization of neurovascular contact is not enough to confirm the presence of NVC [97]. Some neuroradiological features of such contact significantly increase the risk of symptomatic NVC. They include thinning of the nerve, arterial imprint, grooving of the nerve, or distorted course of the nerve [97]. On the other hand, it should be emphasized that the nerve itself can be displaced and changed by one vessel and the symptoms of NVC can be caused by another vessel impinging on the nerve in its REZ.

#### Diffusion Tensor Imaging and Diffusion-Weighted Imaging

Diffusion tensor imaging (DTI) and diffusion-weighted imaging (DWI) are forms of MR imaging that were investigated in patients with NVC [98,99]. The most commonly described alternations were observed in fractional anisotropy (FA) in DTI and apparent diffusion coefficient (ADC) [100,101]. It was reported that the reduction FA and increase in ADC had a close relationship to white matter tract degeneration. Such changes in mentioned parameter values are probably triggered by diffusivity averaging in all spatial directions caused by myelin deficiency [82]. Interestingly, both FA and ADC may be designated also in small anatomical areas. Thanks to this it is possible to evaluate NVCs consequences in these MRI protocols [102].

The usefulness of DTI in NVCs evaluation was mainly assessed on an example of TN [99,102,103,104,105,106]. The majority of studies reported that DTI can reveal alterations (as a sign of anisotropic microstructural changes) of nerve integrity on affected sites compared to non-affected sites and healthy controls [101,103,106]. Moreover, Willsey et al. found that the DTI examination allows for a diagnosis of TN subtypes (TN1 and TN2), thus facilitating patients’ selection for surgical procedures, and can be a prognostic marker following an intervention [105]. An interesting issue was raised in a prospective study on DTI usefulness evaluation performed by Lutz et al. [103]. They reported that in 3 symptomatic cases that were qualified for the MVD procedure preoperative high-resolution MRI did not reveal any NVC occurrence. Interestingly, after the surgery symptoms resolved. In reevaluation retrospectively also there were no NVCs in MRI. Lutz et al. revealed that in mentioned cases with negative MRI, the FA parameter was lower [103].

## 6. Future Directions and Conclusions

### 6.1. Importance of Further Studies in the Field

The SCA is frequently involved in symptomatic NVCs (40–90%). Its variability may be also a cause of HFS. The significance of NVC between SCA and the oculomotor nerve is poorly discussed in the literature. Nevertheless, it seems that in extremely rare cases mentioned NVC can be symptomatic and clinically manifested as ONP or oculomotor NMT. Up to date, little is known about SCA variability in the context of NVC. Further studies are required to analyze the influence of such variations on risk symptomatic NVCs development.

### 6.2. Conclusion and Clinical Points

#### 6.2.1. Conclusion and Summary of Key Points

- Although SCA is a key vessel related to trigeminal neuralgia pathogenesis, the current state of knowledge about the link between SCA variability and NVC (especially TN) occurrence remains limited and required further investigation.
- Neuroimaging enables the proper differential diagnosis in NVC patients, i.e. ruling out brain tumors, aneurysms, arteriovenous malformations, and others.
- The success rate of MVD strongly depends on careful visualization of the nerve in its REZ at surgery not on radiological examination. Many NVCs, that can be observed intraoperatively are not seen in neuroimaging. Visualization of contact between nerve and vessel in the radiological examination without clinical symptoms does not suffice for the diagnosis of NVCs.
- Knowledge about possible anatomical variants of SCA may improve patients’ diagnosis, choosing an appropriate treatment strategy, and patients’ outcome surgical.
- A tortuous anterior pontomesencephalic segment of the SCA may lead to misdiagnosis as it may mimic arteriovenous malformation on imaging.

#### 6.2.2. Clinical Points

- Typical pain in TN is characterized by sharp, electric shock-like paroxysmal lancinating pain, lasting a few seconds, typically triggered by sensory stimulation. In these cases, the neurologic exam remains normal, except for a mild sensory loss. A pain of another character situated in the trigeminal nerve innervation zone is known as atypical facial pain.
- Although the majority of the TN patients show a good response to carbamazepine, there are those (especially refractory cases) in whom microvascular decompression should be considered.

## Data Availability

Not applicable.

## Supplementary Materials

The following supporting information can be downloaded at: https://www.mdpi.com/article/10.3390/biomedicines11072009/s1, Figure S1: The angiography with left superior cerebellar artery aneurysm embolization: (A) angiography revealed aneurysm (7 × 6 × 5 mm in size), (B,C) the aneurysm was further embolized with MicroPlex Coil System, Figure S2: The angiography with left superior cerebellar artery aneurysm embolization: (A) The angiography revealed aneurysm, (B,C) the aneurysm was embolized with MicroPlex Coil System.

## Abbreviations

ADC	apparent diffusion coefficient
AICA	anterior inferior cerebellar artery
BA	basilar artery
DTI	diffusion tensor imaging
FA	fractional anisotropy
HFS	hemifacial spasm
MRI	magnetic resonance imaging
MVD	microvascular decompression
N.D.	no data
NMT	neuromyotonia
NVC	neurovascular compression
ONP	oculomotor nerve palsy
PCA	posterior cerebral artery
PICA	posterior inferior cerebellar artery
REZ	root entry/exit zone
SCA	superior cerebellar artery
VA	vertebral artery
