# Supplementary material for: The Superior Cerebellar Artery: Variability and Clinical Significance
Source: Biomedicines. 2023 Jul 17;11(7):2009. doi: 10.3390/biomedicines11072009 (PMC10376954; doi:10.3390/biomedicines11072009)
Supplement: Supplementary file 1 [file biomedicines-11-02009-s001.zip › biomedicines-2488784-supplementary.pdf]

biomedicines

MDPI

Supplementary materials

The Superior Cerebellar Artery: Variability and Clinical Significance

1. Aneurysm of superior cerebellar artery: a case study (1)

A 82-year-old righthanded lady was referred to the Department of Neurosurgery and Neuro-Oncology with the sudden onset of severe headache that took place a day before. A CT scan showed subarachnoid hemorrhage (SAH), while CTA revealed a left superior cerebellar artery aneurysm. On admission, she was drowsy and disoriented in time, place, and herself; GCS 13 (E3, V4, M6), grade III on the Hunt and Hess scale. There was no meningeal irritation nor limb weakness. There were signs of CN IX and CN X damage on the left side. Past medical history: ischemic heart disease, diabetes mellitus type 2, mixed hyperlipidemia, and atherosclerosis. Endovascular treatment was chosen. The embolization was performed on the next day (see **Figure 1A** – before, **1B, C** - after). Although there was no complication of the procedure, the patient developed a delayed ischaemic neurological deficit, and progressive deterioration of general status was observed over the following days. Despite intensive treatment employed, her condition deteriorated and the patient expired on the sixth day after the admission.

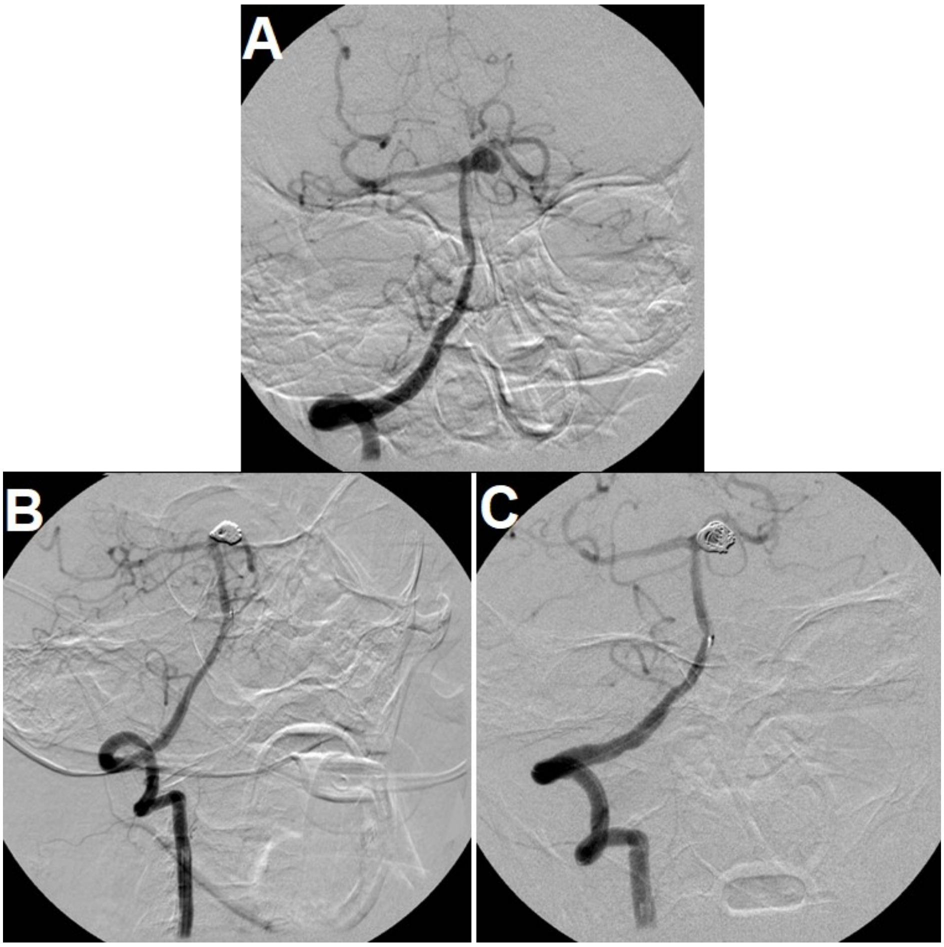

Figure S1. The angiography with left superior cerebellar artery aneurysm embolization: **A)** angiography revealed aneurysm (7x6x5mm in size), **B, C)** the aneurysm was further embolized with MicroPlex Coil System.

2. Aneurysm of superior cerebellar artery: a case study (2)

A 76-year-old righthanded lady was referred to the Department of Neurosurgery and Neuro-Oncology with a sudden onset of severe headache. A CT scan confirmed massive SAH presumably from a ruptured aneurysm of the left SCA, while CTA revealed an aneurysm of the basilar artery at the origin of the left SCA. On admission, she was drowsy and disoriented in time, place, and herself; GCS 13 (E3, V4, M6), grade III on the Hunt and Hess scale. Her past medical history was unknown. Endovascular treatment was chosen. The embolization was performed four days later (see Figure S2). After the procedure, transient right-sided paresis, eye movement disorders, and increased drowsiness were observed. Symptoms resolved with medical treatment. On the next day, bleeding from the lower gastrointestinal tract was observed. Conservative management, was recommended by general surgeons. Four days after the embolization, there was neurological deterioration. Control CT scans showed progression of ischemic foci in the cerebellum and the left hemisphere. She died 10 days after the embolization.

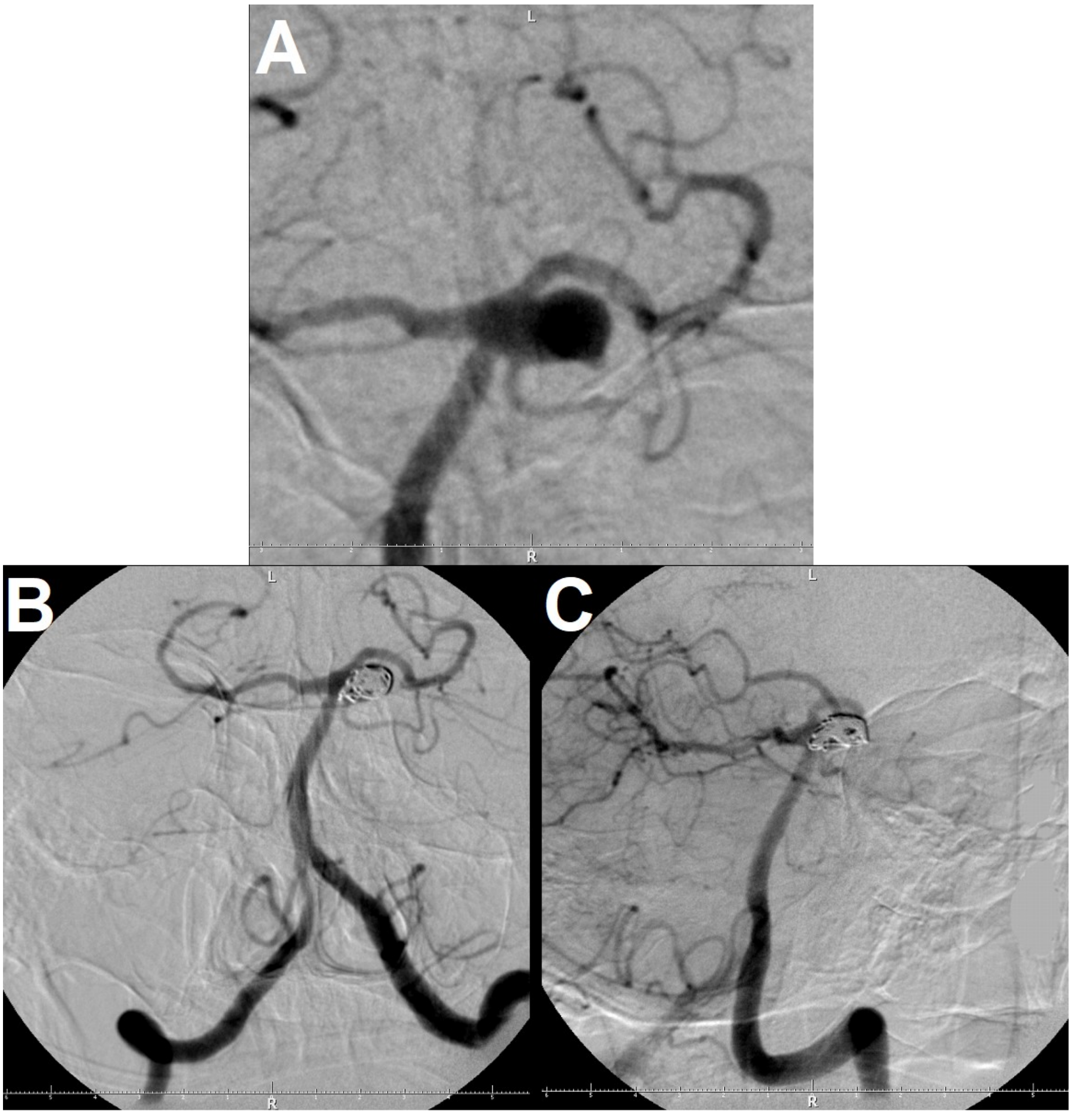

Figure S2. The angiography with left superior cerebellar artery aneurysm embolization: **A)** The angiography revealed aneurysm, **B, C)** the aneurysm was embolized with MicroPlex Coil System.
